# Automated wound care by employing a reliable U-Net architecture combined with ResNet feature encoders for monitoring chronic wounds

**DOI:** 10.3389/fmed.2024.1310137

**Published:** 2024-01-31

**Authors:** Maali Alabdulhafith, Abduljabbar S. Ba Mahel, Nagwan Abdel Samee, Noha F. Mahmoud, Rawan Talaat, Mohammed Saleh Ali Muthanna, Tamer M. Nassef

**Affiliations:** ^1^Department of Information Technology, College of Computer and Information Sciences, Princess Nourah bint Abdulrahman University, Riyadh, Saudi Arabia; ^2^School of Life Science, University of Electronic Science and Technology of China, Chengdu, China; ^3^Rehabilitation Sciences Department, Health and Rehabilitation Sciences College, Princess Nourah bint Abdulrahman University, Riyadh, Saudi Arabia; ^4^Biotechnology and Genetics Department, Agriculture Engineering, Ain Shams University, Cairo, Egypt; ^5^Institute of Computer Technologies and Information Security, Southern Federal University, Taganrog, Russia; ^6^Computer and Software Engineering Department, Engineering College, Misr University for Science and Technology, 6th of October, Egypt

**Keywords:** chronic wound, transfer learning, segmentation, UNet, ResNet34

## Abstract

Quality of life is greatly affected by chronic wounds. It requires more intensive care than acute wounds. Schedule follow-up appointments with their doctor to track healing. Good wound treatment promotes healing and fewer problems. Wound care requires precise and reliable wound measurement to optimize patient treatment and outcomes according to evidence-based best practices. Images are used to objectively assess wound state by quantifying key healing parameters. Nevertheless, the robust segmentation of wound images is complex because of the high diversity of wound types and imaging conditions. This study proposes and evaluates a novel hybrid model developed for wound segmentation in medical images. The model combines advanced deep learning techniques with traditional image processing methods to improve the accuracy and reliability of wound segmentation. The main objective is to overcome the limitations of existing segmentation methods (UNet) by leveraging the combined advantages of both paradigms. In our investigation, we introduced a hybrid model architecture, wherein a ResNet34 is utilized as the encoder, and a UNet is employed as the decoder. The combination of ResNet34’s deep representation learning and UNet’s efficient feature extraction yields notable benefits. The architectural design successfully integrated high-level and low-level features, enabling the generation of segmentation maps with high precision and accuracy. Following the implementation of our model to the actual data, we were able to determine the following values for the Intersection over Union (IOU), Dice score, and accuracy: 0.973, 0.986, and 0.9736, respectively. According to the achieved results, the proposed method is more precise and accurate than the current state-of-the-art.

## 1 Introduction

Rehabilitation therapists assume a crucial role in the home-based therapy of injuries caused by pressure and other types of wounds. Therapists possess a robust understanding of evidence-based practices in the field of wound care. Through collaborative efforts with the interdisciplinary care team, therapists are capable of offering valuable assistance in the management of wound care, hence contributing to its effectiveness. Rehabilitation therapists receive specialized training in wound assessment and documentation and provide critical interventions to improve patient outcomes and the financial sustainability of home healthcare businesses. Physical therapists develop optimal wound treatment plans, including restoring mobility, strengthening, and healing. A wound is considered chronic if healing takes more than 4 weeks without progress (1). There are a variety of things that can impede the usual process of wound healing. People who have comorbidities like diabetes and obesity are more likely to suffer from wounds like these. The care for these injuries comes at a very high financial cost. Patients who are suffering from chronic wounds require more intensive wound care as opposed to patients who are suffering from acute wounds (2). They have to visit a doctor on a consistent basis so the doctor can monitor how well the wound is healing. The management of wounds needs to adhere to the best practices available in order to facilitate healing and reduce the risk of wound complications. The evaluation of wound healing begins with a thorough assessment of the wound. When determining the rate of wound healing, one of the most important aspects of wound care practice is the utilization of clinical standards (3). These guidelines prescribe the usual documentation of wound-related information, such as wound color, size, and composition of wound tissue (4). The conventional technique involves professionals in the area of treating wounds to take measurements of the wound area as well as its tissue structure. This method is labor-intensive, expensive, and difficult to replicate (5, 6). In this study, we are introducing an approach for automatically assessing wounds that makes use of automatic wound color segmentation in addition to algorithms that are based on artificial intelligence.

Wound healing is a complex and dynamic biological process that results in tissue regeneration, restoration of anatomical integrity, and restoration of similar functionality (7). According to assumptions, the advanced wound care market is anticipated to surpass a value of $22 billion by the year 2024 (5). This discrepancy may be attributed to the rise in outpatient wound treatments that are presently being administered (8). Chronic wounds are categorized as wounds that have exceeded the typical healing timeline and remain open for a duration surpassing 1 month (9). Chronic wound infections have been found to cause substantial morbidity and make a significant contribution to the rising costs of healthcare (10). The development of advanced wound care technologies is imperative in order to address the increasing financial strain on national healthcare budgets caused by chronic wounds, as well as the significant adverse effects these wounds have on the quality of life of affected patients (11). Currently, there is a significant prevalence of patients experiencing wound infections and chronic wounds. The management of postoperative wounds continues to present a laborious and formidable task for healthcare professionals and individuals undergoing surgery. There exists a significant need for the advancement of a collection of algorithms and associated methodologies aimed at the timely identification of wound infections and the autonomous monitoring of wound healing progress (12, 13). A pressure ulcer, in accordance with the European Pressure Ulcer Advisory Panel, is characterized as a specific region of restricted harm to both the underlying tissue as well as the skin, resulting from an action of pressure, shear, and friction. A pressure ulcer is classified as a chronic injury resulting from persistent and prolonged soft tissue compression compared to a bony prominence, as well as a rigid surface or medical equipment (14). The occurrence of diabetic foot ulcer (DFU) represents a significant complication associated with the presence of diabetes (15). DFU is the primary factor contributing to limb amputations. In line with the World Health Organization (WHO), it has been estimated that approximately 15% of individuals diagnosed with diabetes mellitus experience the occurrence of DFU at least once throughout their lifespan (16). Image segmentation is an essential task when it comes to computer vision and image processing (17). The process of image segmentation holds significant importance in numerous medical imaging applications as it aids in automating or facilitating the identification and drawing of lines around essential regions of interest and anatomical structures (18). Nevertheless, it is challenging to generalize the performance of segmentation across various wound images. The presence of various wound types, colors, shapes, body positions, background compositions, capturing devices, and image-capturing conditions contributes to the considerable diversity observed in wound images (19). Wound segmentation in medical imaging has advanced with hybrid models. Relevant studies (20–22) emphasize community-driven chronic wound databases, telemedicine-based frameworks, and M-Health for tele-wound monitoring.CNNs, ensemble learning, attention mechanisms, and transfer learning improve crop and rice disease detection and breast cancer classification (23–26). Our research builds on these findings to develop a hybrid model for improved chronic wound segmentation accuracy. The objective of our study is to concentrate on the advancement of a deep-learning methodology for wound segmentation. We suggest a unique framework that integrates the advantageous features of the UNet architecture and the ResNet34 model in order to enhance the efficacy of image segmentation tasks.

The main contribution of this study is the development and evaluation of a hybrid model for wound segmentation that seamlessly integrates advanced deep learning approaches with traditional image processing methods. This innovative alliance aims to improve the accuracy and reliability of wound segmentation significantly, overcoming limitations identified in existing methodologies.

The article’s remaining sections are arranged in the manner shown below. Related works: Detailed review of previous research in the field. Materials and Methods: Describes the used dataset, the architecture and implementation of the hybrid model, detailing how advanced deep learning techniques integrate with traditional image processing techniques. Results: Presents the results of the experimental analysis evaluating the performance of the hybrid model. Discussion: Analyzes the results, discusses their implications, compares them with existing segmentation methods, and explores potential applications of the hybrid model in clinical practice. Conclusions: Summarizes the main contributions, highlights the importance of the developed hybrid model, and suggests directions for future research.

## 2 Related work

Wang et al. (27) proposed the implementation of an integrated system that automates the process of segmenting wound regions and analyzing wound conditions in images of wounds. In contrast to previous segmentation techniques that depend on manually designed features or unsupervised methods, the study’s authors introduce a deep learning approach that simultaneously learns visual features relevant to the task and carries out wound segmentation. In addition, acquired features are utilized for subsequent examination of wounds through two distinct approaches: identification of infections and forecasting of healing progress. The proposed methodology demonstrates computational efficiency, with an average processing time of under 5 s per wound image of dimensions 480 by 640 pixels when executed on a standard computer system. The evaluations conducted on a comprehensive wound database provide evidence supporting the efficacy and dependability of the proposed system. Ohura et al. (28) established several convolutional neural networks (CNNs) using various methods and architectural frameworks. The four architectural models considered in their study were LinkNet, SegNet, U-Net, and U-Net with the VGG16 Encoder Pre-Trained on ImageNet (referred to as Unet_VGG16). Every convolutional neural network (CNN) was trained using supervised data pertaining to sacral pressure ulcers (PUs). The U-Net architecture yielded the most favorable outcomes among the four architectures. The U-Net model exhibited the second-highest level of accuracy, as measured by the area under the curve (AUC) with a value of 0.997. Additionally, it demonstrated a high level of specificity (0.943) and sensitivity (0.993). Notably, the highest values were achieved when utilizing the Unet_VGG16 variant of the U-Net model. The architecture of U-Net was deemed to be the most practical and superior compared to other architectures due to its faster segmentation speed in comparison to Unet_VGG16. Scebba et al. (19) introduced the detect-and-segment (DS) method, which is a deep learning technique designed to generate wound segmentation maps that possess excellent generalization skills. The proposed methodology involved the utilization of specialized deep neural networks to identify the location of the wound accurately, separate the wound from the surrounding background, and generate a comprehensive map outlining the boundaries of the wound. The researchers conducted an experiment in which they applied this methodology to a dataset consisting of diabetic foot ulcers. They then proceeded to compare the results of this approach with those obtained using a segmentation method that relied on the entire image. In order to assess the extent to which the DS approach can be applied to data that falls outside of its original distribution, the researchers evaluated its performance on four distinct and independent data sets. These additional data sets encompassed a wider range of wound types originating from various locations on the body. The Matthews’ correlation coefficient (MCC) exhibited a notable enhancement, increasing from 0.29 (full image) to 0.85 (DS), as observed in the analysis of the data set for diabetic foot ulcers. Upon conducting tests on the independent data sets, it was observed that the mean Matthews correlation coefficient (MCC) exhibited a significant increase from 0.17 to 0.85. In addition, the utilization of the DS facilitated the segmentation model’s training with a significantly reduced amount of training data, resulting in a noteworthy decrease of up to 90% without any detrimental effects on the segmentation efficiency. The proposed data science (DS) approach represents a significant advancement in the automation of wound analysis and the potential reduction of efforts required for the management of chronic wounds. Oota et al. (29) constructed segmentation models for a diverse range of eight distinct wound image categories. In this study, the authors present WoundSeg, an extensive and heterogeneous dataset comprising segmented images of wounds. The complexity of segmenting generic wound images arises from the presence of heterogeneous visual characteristics within images depicting similar types of wounds. The authors present a new image segmentation framework called WSNet. This framework incorporates two key components: (a) wound-domain adaptive pretraining on a large collection of unlabelled wound images and (b) a global-local architecture that utilizes both the entire image and its patches to capture detailed information about diverse types of wounds. The WoundSeg algorithm demonstrates a satisfactory Dice score of 0.847. The utilization of the existing AZH Woundcare and Medetec datasets has resulted in the establishment of a novel state-of-the-art. Buschi et al. (30) proposed a methodology to segment the pet wound images automatically. This approach involves the utilization of transfer learning (TL) and active self-supervised learning (ASSL) techniques. Notably, the model is designed to operate without any manually labeled samples initially. The efficacy of the two training strategies was demonstrated in their ability to produce substantial quantities of annotated samples without significant human intervention. The procedure, as mentioned earlier, enhances the efficiency of the validation process conducted by clinicians and has been empirically demonstrated to be an effective strategy in medical analyses.The researchers discovered that the EfficientNet-b3 U-Net model, when compared to the MobileNet-v2 U-Net model, exhibited superior performance and was deemed an optimal deep learning model for the ASSL training strategy. Additionally, they provided numerical evidence to support the notion that the intricacy of wound segmentation does not necessitate the utilization of intricate, deep-learning models. They demonstrated that the MobileNet-v2 U-Net and EfficientNet-b3 U-Net architectures exhibit comparable performance when trained on a bigger collection of annotated images. The incorporation of transfer learning components within the ASSL pipeline serves to enhance the overall ability of the trained models to generalize. Rostami et al. (31) developed an ensemble-based classifier utilizing deep convolutional neural networks (DCNNs) to effectively classify wound images into various classes, such as surgical, venous ulcers, and diabetic. The classification scores generated by two classifiers, specifically the patch-wise and image-wise classifiers, are utilized as input for a multilayer perceptron in order to enhance the overall classification performance. A 5-fold cross-validation strategy is used to evaluate the suggested method. The researchers achieved the highest and mean classification accuracy rates of 96.4% and 94.28%, respectively, for binary classification tasks. In contrast, for 3-class classification problems, they attained maximum and average accuracy rates of 91.9% and 87.7%, respectively. The classifier under consideration was evaluated against several widely used deep classifiers and demonstrated notably superior accuracy metrics. The proposed method was also evaluated on the Medetec wound image dataset, yielding accuracy values of 82.9% and 91.2% for 3-class and binary classifications, respectively. The findings indicate that the method proposed by the researchers demonstrates effective utility as a decision support system for wound image classification and other clinically relevant applications. Huang et al. (32) proposed an innovative approach to automatically segment and detect wounds by leveraging the Mask R-CNN framework. Their study employed a dataset comprising 3,329 clinical wound images, encompassing wounds observed in patients diagnosed with peripheral artery disease, as well as those resulting from general trauma. The implementation of the Mask R-CNN framework was utilized for the purpose of detecting and distinguishing wounds. The outcomes of their methodology were noteworthy, as evidenced by an Intersection over Union score of 0.69, precision rate of 0.77, recall rate of 0.72, average precision of 0.71 and *F*1 score of 0.75. The metrics as mentioned above serve as indicators of the precision and efficacy of the suggested framework for the segmentation and diagnosis of wounds. Foltynski et al. (33) described an automated service for measuring wound areas, which enables accurate measurements by employing adaptive calibration techniques specifically designed for curved surfaces. The deep learning model, which utilized convolutional neural networks (CNNs), underwent training with a dataset consisting of 565 wound images. Subsequently, the model was employed for image segmentation, specifically to discern the wound area and calibration markers. The software that has been developed is capable of calculating the area of a wound by utilizing the pixel count within the wound region, as well as a calibration coefficient derived from the measured distances between ticks located at calibration markers. The outcome of the measurement is transmitted to the user via the designated email address. The wound models exhibited a median relative error of 1.21% in the measurement of wound area. The effectiveness of the convolutional neural network (CNN) model was evaluated on a total of 41 actual wounds and 73 simulated wound models. The mean values for the accuracy, specificity, Intersection over Union, and dice similarity coefficient in the context of wound identification were found to be 99.3%, 99.6%, 83.9%, and 90.9%, respectively. The efficacy of the service has been demonstrated to be high, making it suitable for monitoring wound areas. Pereira et al. (34) developed a comprehensive system that includes a deep learning segmentation model called MobileNet-UNet. This model is capable of identifying the specific area of a wound and classifying it into one of three categories: chest, drain, or leg. Additionally, the system incorporates a machine learning classification model that utilizes different algorithms (support vector machine, k-nearest neighbors, and random forest) to predict the likelihood of wound alterations for each respective category (chest, leg, and drain). The deep learning model performs image segmentation and classifies the wound type. Subsequently, the machine learning models employ classification techniques to categorize the images based on a set of color and textural features that are obtained from the output region of interest. These features are then utilized to feed into one of the three wound-type classifiers, ultimately leading to a final binary decision regarding the alteration of the wound. The segmentation model attained a mean average precision (AP) of 90.1% and a mean Intersection over Union (IoU) of 89.9%. The utilization of distinct classifiers for the final classification yielded greater efficacy compared to employing a single classifier for all different kinds of wounds. The classifier for leg wounds demonstrated superior performance, achieving an 87.6% recall rate and 52.6% precision rate.

## 3 Materials and methods

### 3.1 Dataset

**Figure 1:**
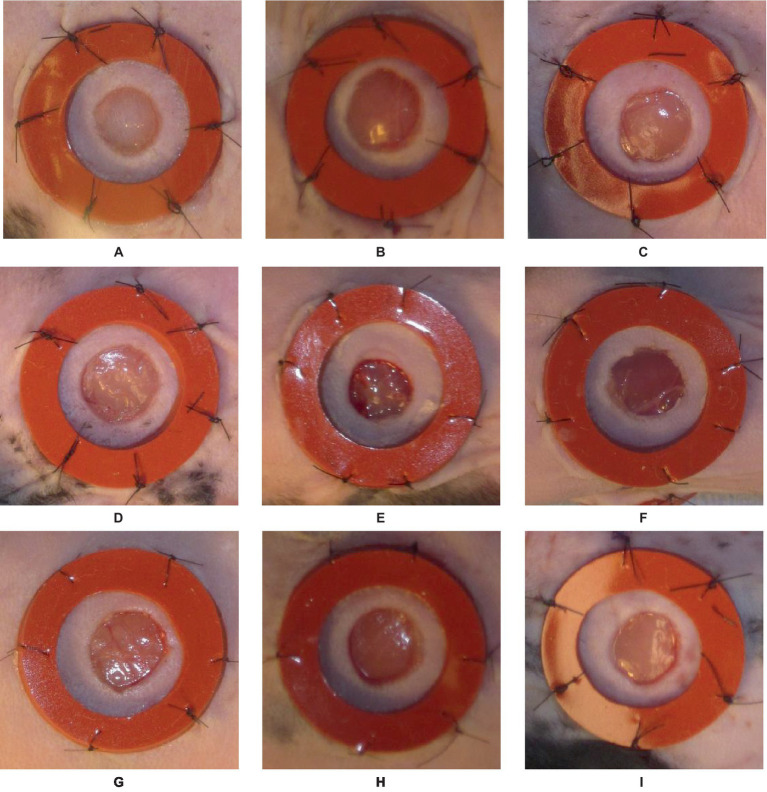
An illustration of nine different samples **(A–I)** from the data set used in the study, presented in visual form for more detailed analysis and understanding.

The initial dataset comprises around 256 images of laboratory mice with inflicted wounds (35). The study encompasses a total of eight mice, with four mice assigned to Cohort 1 (C1) and four mice assigned to Cohort 2 (C2). The observation period spans 16 days, during which the healing process is monitored. The two cohorts are indicative of two separate experimental conditions, thus necessitating the grouping of results based on cohort. Each mouse in the study exhibits a pair of wounds, one located on the left side and the other on the right side. Consequently, the dataset comprises a collection of time series data encompassing a total of 16 individual wounds. Every wound is bounded by a circular cast that encompasses the injured region. The inner diameter of this splint measures 10 mm, while the outer diameter measures 16 mm. The splint is utilized as a reference object in the workflow due to its fixed inner and outer diameter, providing a known size. It is essential to acknowledge that a significant proportion, precisely over 25%, of the images within this dataset exhibit either missing casts or substantial damage to the splints. The images exhibit various challenges, including variations in image rotation (portrait vs. landscape), ranging lighting conditions, inaccurate or obscured tape measure position, multiple visible wounds, significant occlusion of the wound, and the relative positioning of the wound within the picture frame. The challenges mentioned above prompted researchers to explore the integration of deep learning algorithms with conventional image processing techniques. The approach of pre-processing for wound segmentation involves multiple steps. The images are acquired from the dataset containing photographs of wounds. Subsequently, the labelme tool is employed to designate the wound area and the encompassing region. Data augmentation techniques such as vertical and horizontal flips, transpose, and rotation are employed. The inclusion of diverse data and the subsequent preparation of the dataset were crucial steps in enhancing its quality and suitability for training an effective wound segmentation model. Different samples from the described above dataset are presented in Figure 1.

### 3.2 Data preprocessing

**Figure 2:**
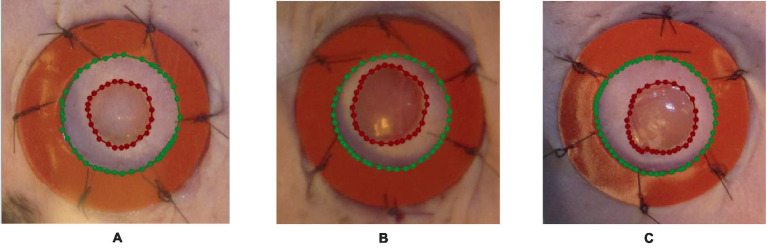
An illustration of the outcomes obtained from the process of data labeling utilizing the labelme tool, where the blue polynomial line in **A–C** outcomes indicates the area around the wound, while the red polynomial line indicates the wounded area.

The pre-processing of data is of most tremendous significance in the preparation of input data for machine learning tasks. In the context of wound segmentation, it is imperative to perform image pre-processing in order to improve the quality of the images, extract pertinent features, and augment the dataset to enhance the performance of the model. This section will examine the different stages encompassed in the process of data pre-processing for wound segmentation. In brief, the process of data pre-processing for wound segmentation entails several steps. Firstly, the images are obtained from the wound photo dataset. Next, the wound area and the surrounding region are labeled using the labelme library in Python. Lastly, data augmentation methods such as vertical and horizontal flips, rotation, and transpose are applied. The following steps are essential in improving the dataset, enhancing its diversity, and preparing it for the training of a resilient wound segmentation model. Firstly, the images are obtained from the dataset containing photographs of wounds (35). The images function as the primary source of input for the segmentation task. The dataset comprises a compilation of images portraying various categories of wounds. It is necessary to perform image processing and labeling on each image in order to differentiate the injured area from the surrounding region. The labelme (36) library is employed for image annotation and labeling in the Python programming language. The labelme library offers a user-friendly graphical interface that allows users to annotate regions of interest within images. In order to facilitate segmentation, distinct classes are created by applying separate labels to both the wound area and the surrounding area. Figure 2 illustrates various instances of data labeling.

The next step is data augmentation after the labeling procedure is finished. Data augmentation techniques are utilized to enhance the diversity and quantity of the dataset, thereby potentially enhancing the model’s generalization capability. When it comes to wound segmentation, several frequently utilized data augmentation techniques encompass horizontal and vertical flips, rotation, and transposition. The transformations of vertical and horizontal flips involve mirroring the image along the horizontal or vertical axis, respectively. These operations induce variations in the dataset by altering the orientation of the wounds. The process of rotation entails the application of a specific angle to the image, thereby emulating diverse viewpoints of the wound. The operation of transposition involves a straightforward flipping of an image along its diagonal axis.

**Figure 3:**
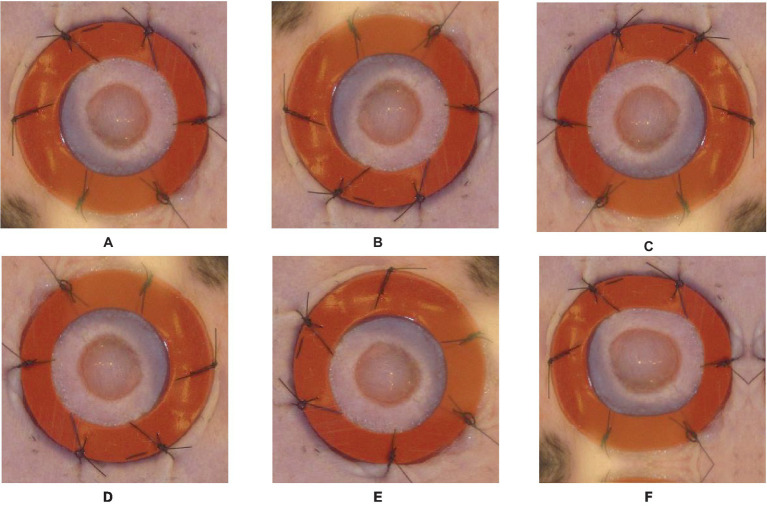
Original image and Augmented images with an illustration for each process done on the original image: **(A)**—original image, **(B)**—vertical flip, **(C)**—horizontal flip, **(D)**—random rotate 90, **(E)**—transpose, **(F)**—grid distortion.

By implementing these data augmentation techniques on the labeled images, we produce supplementary training instances that exhibit variations in orientation and position. The augmented dataset utilized in this study encompasses a broader spectrum of potential scenarios, thereby enhancing the model’s capacity to acquire more resilient features and enhance its precision in accurately segmenting wounds. To retain their relationship with the augmented images, the labels must be altered appropriately during the data augmentation process. In the event that an image undergoes horizontal flipping, the associated labels must undergo a corresponding horizontal flipping as well. Figure 3 provides an example of the augmentation process, illustrating the original image and the augmentation steps applied to it.

### 3.3 Proposed framework

**Figure 4:**
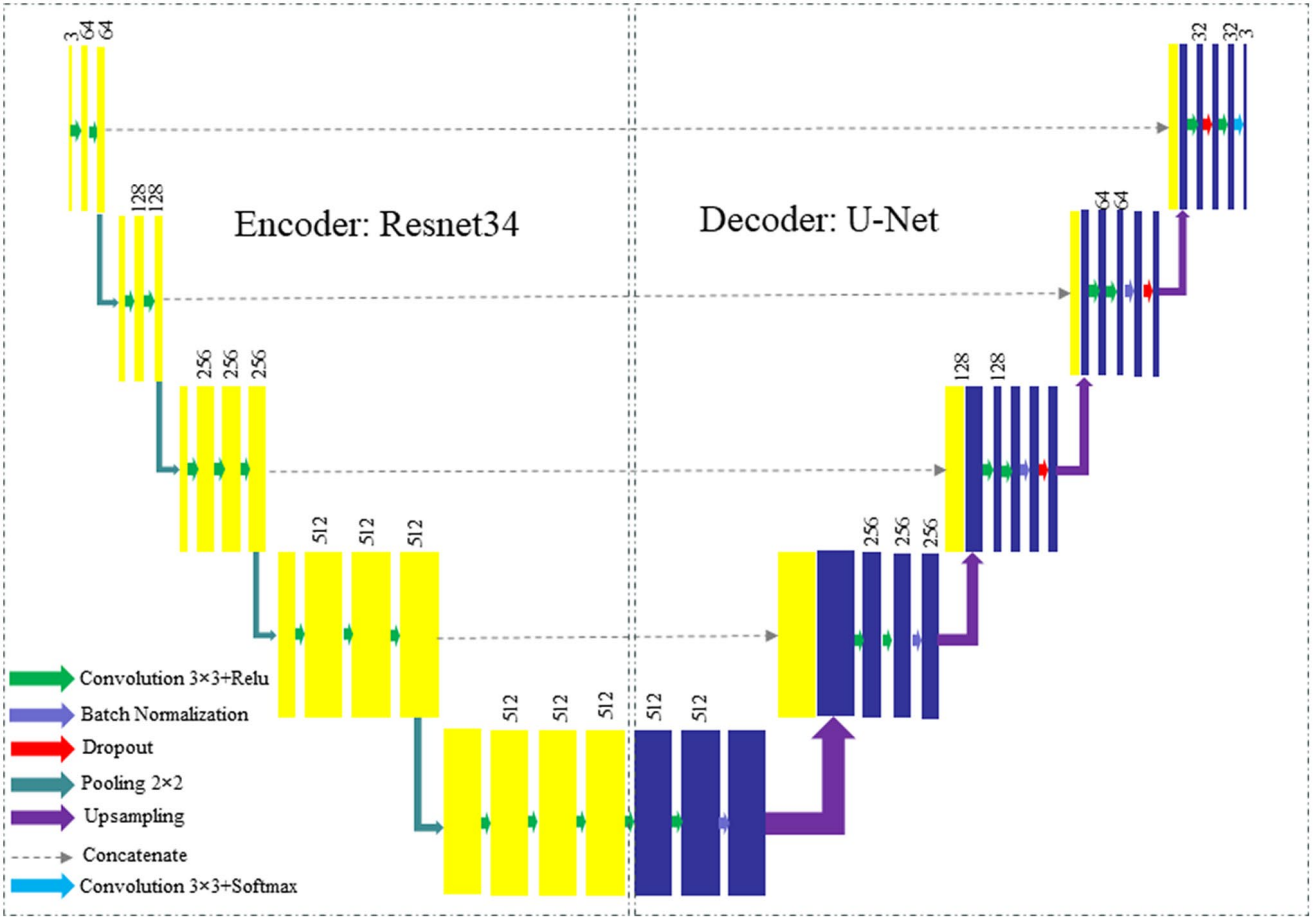
The U-Net architecture that is being suggested with a ResNet-34-based encoder.

**Figure 5:**
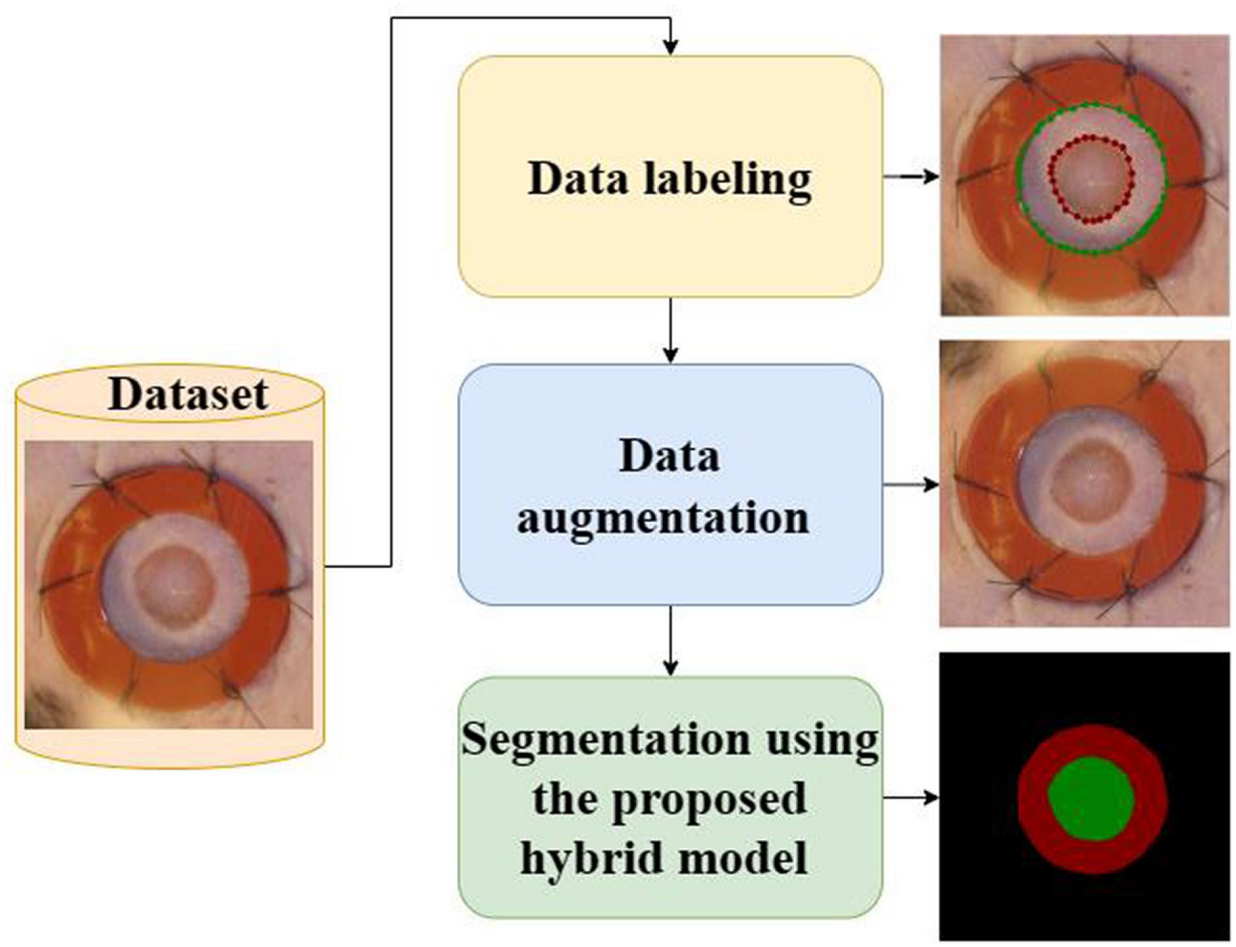
The main steps of the proposed system.

This study introduces a novel framework that integrates the U-Net (37) architecture and the ResNet34 (38) model in order to enhance the efficacy of image segmentation tasks. The proposed framework utilizes the encoding capabilities of ResNet34 as the primary encoder while incorporating the decoder architecture of U-Net to achieve precise and comprehensive segmentation. The diagram depicting the proposed framework is presented in Figure 4, while Figure 5 highlights the main steps of the proposed system.

The framework integrates the robust encoding capabilities of ResNet with the precise and comprehensive segmentation capabilities of U-Net. Through the strategic utilization of the inherent advantages offered by both architectures, our hybrid model endeavors to enhance the efficacy of image segmentation tasks. The integration of fusion and skip connections facilitates a seamless connection between the encoder and decoder, enabling efficient information transmission and accurate segmentation. The framework that has been proposed presents a promising methodology for tackling the challenges associated with image segmentation. It holds the potential to advance the current state-of-the-art in this particular domain significantly. The main elements within the proposed framework may be enumerated as follows:

### 3.4 Encoder-decoder architecture

The proposed framework uses a hybrid model architecture with a ResNet34 as the encoder and a U-Net as the decoder. The integration of ResNet34’s deep representation learning and U-Net’s efficient feature extraction enables us to derive significant advantages. The utilization of the encoder-decoder architecture has been widely recognized as an effective strategy in the context of image segmentation tasks. This architectural design effectively captures and incorporates both low-level and high-level features, thereby facilitating the generation of precise and accurate segmentation maps.

### 3.5 ResNet encoder

The ResNet34, which is an abbreviation for Residual Network 34, is a highly prevalent deep learning framework renowned for its efficacy in addressing the challenge of vanishing gradients in deep neural networks. The encoder employed in our study is a pre-trained ResNet model, which has undergone training on a comprehensive image classification task to acquire extensive and distinctive features. The ResNet encoder is responsible for taking the input image and iteratively encoding it into a compressed feature representation.

### 3.6 U-Net decoder

The U-Net architecture was initially introduced for the segmentation of biomedical images, with the specific aim of generating precise and comprehensive segmentation maps. The system comprises a decoder that facilitates the restoration of spatial information that was lost during the encoding phase. The decoder in the U-Net architecture is composed of a sequence of up sampling and concatenation operations, which progressively restore the initial image resolution while integrating high-level feature maps obtained using the encoder.

### 3.7 Fusion and skip connections

In order to facilitate efficient transmission of information between the encoder and decoder, our hybrid framework integrates fusion and skip connections. The fusion connections facilitate the integration of feature maps derived from the ResNet encoder with their corresponding feature maps in the U-Net decoder. The integration of both low-level and high-level features enables the decoder to enhance the accuracy of the segmentation. Skip connections are utilized to establish direct connections between the encoder and decoder at various spatial resolutions. These connections play an important role in facilitating the transfer of intricate details and spatial information between various layers, thereby enabling the achievement of precise segmentation.

### 3.8 Training and optimization

The introduced framework is trained in a supervised fashion utilizing a dataset that has been annotated with segmentation masks at the pixel level. A suitable loss function, such as the Dice coefficient or cross-entropy, is utilized to quantify the dissimilarity between the ground truth and the predicted segmentation maps. The optimization of network parameters is performed using the Adam algorithm, a gradient-based optimization technique, along with a suitable schedule for the learning rate.

## 4 Results

This section presents the results of a study aimed at segmenting the wound using the developed hybrid Resent 34 and U-Net model. The research process included the development of the model, data augmentation, training of the model on these data, and testing on actual data not involved in the training process. As a result, the following were obtained:

### 4.1 Development of an algorithm for segmentation of the wound

An algorithm was developed based on a combination of the Resent34 neural network and U-NET architecture. The algorithm consists of several stages, including preliminary data labeling, processing, and building and training of the model. Preliminary processing includes scaling and normalization of the data to improve the quality and speed of the model’s training.

### 4.2 Model training

To train the model, a set of data was used, including images of wounds with labeling segmentation. The model was optimized using Adam optimizer, and then the training was carried out on a 12th Gen Intel^®^ i7.

### 4.3 Testing on real data

After training, the model was tested on actual data that were not used in the learning process. For each image of the wound, the model predicted a segmentation mask indicating the wounded area. Metrics were used, such as Intersection over Union (IOU) and Dice coefficient to assess the quality of the segmentation.

#### 4.3.1 Intersection over Union

The IOU metric is used to assess the similarity between the predicted mask and the actual mask of the wound. IOU is calculated by dividing the intersection between the two masks into the area of their association. The higher IOU indicates the best segmentation.

#### 4.3.2 Dice coefficient

The Dice coefficient is also used to measure similarities between the predicted mask and the actual mask of the wound. It is calculated as the double value of the intersection area, divided into the sum of the areas of predicted and actual masks. The high value of the Dice coefficient also indicates a more accurate segmentation.

**Figure 6:**
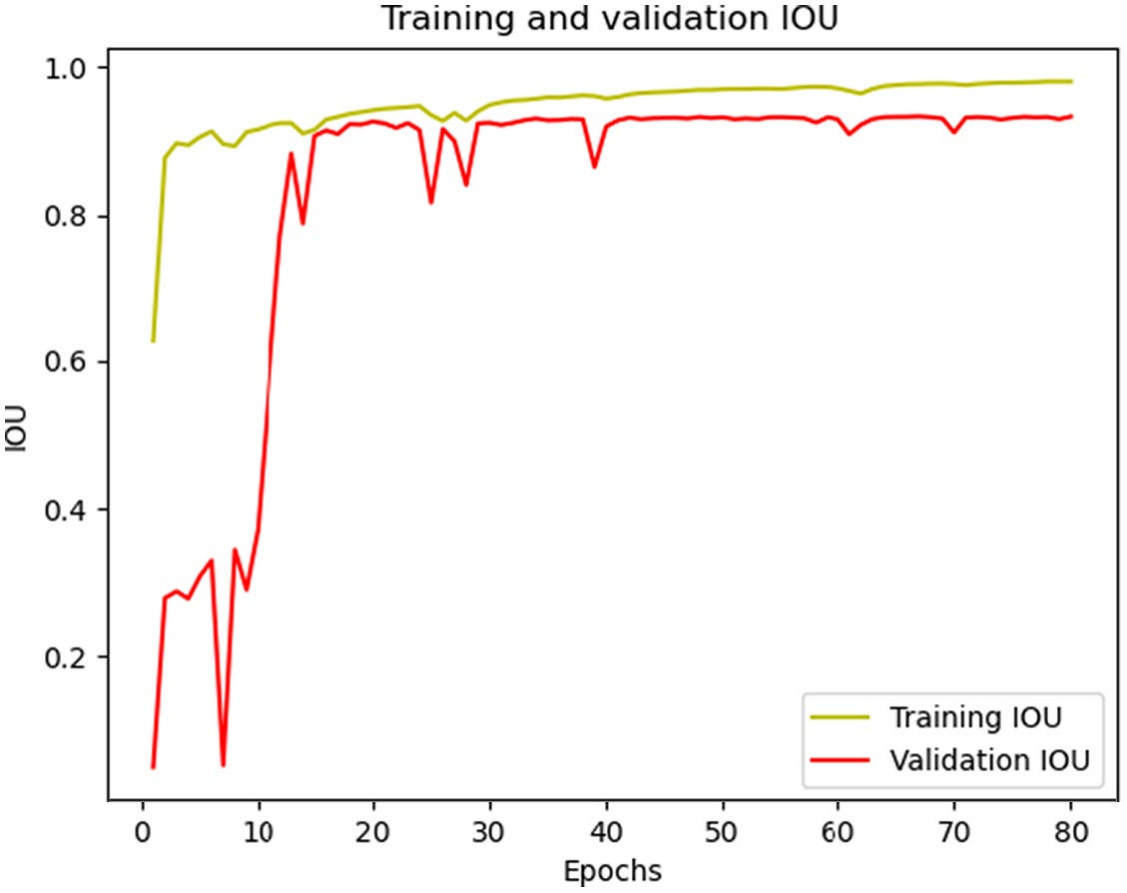
Training and validation IOU curves.

**Figure 7:**
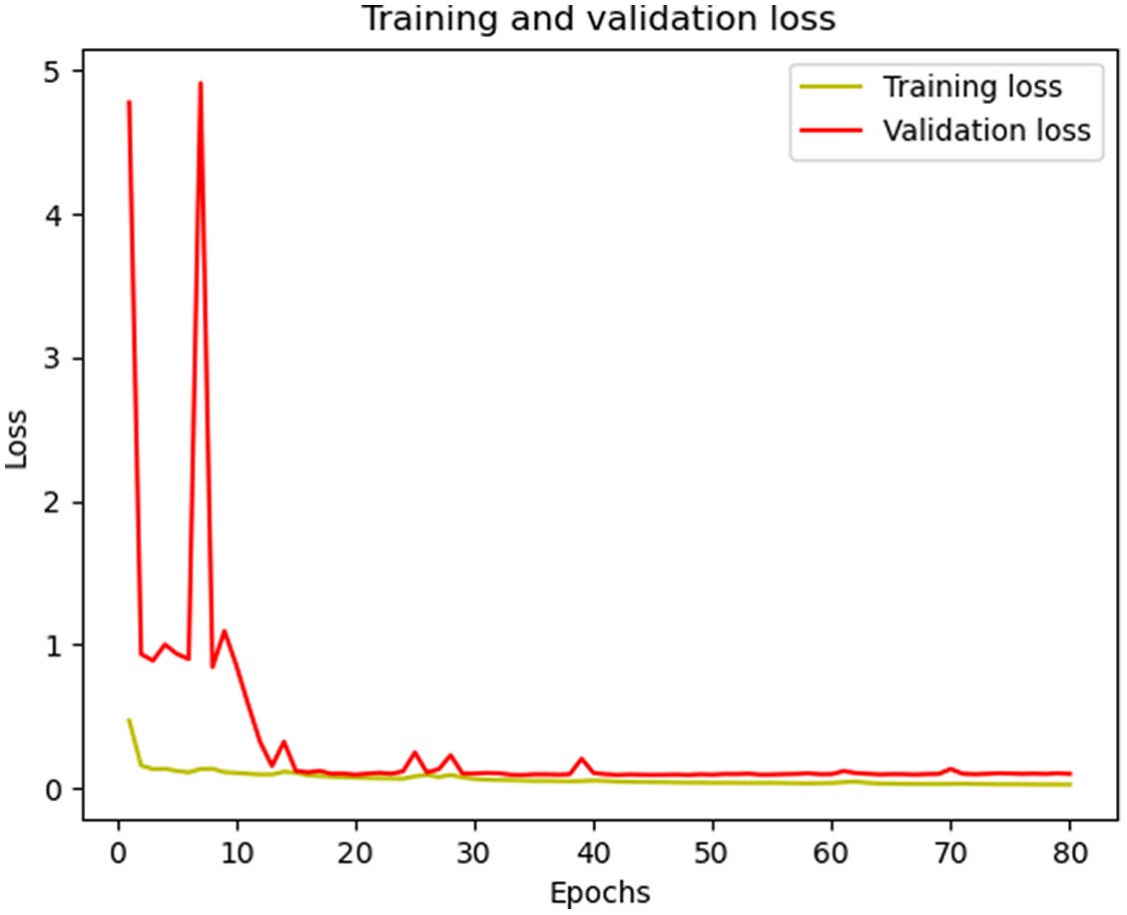
Training and validation loss curves.

As a result of testing the model on the actual data, the following values of the IOU, Dice metrics, and accuracy were obtained: 0.973, 0.986, and 0.9736, respectively. These results confirm that the developed wound segmentation algorithm achieves good results and demonstrates a high degree of similarity between the wound-predicted and actual masks. High values of the IOU and Dice metrics indicate the accuracy and quality of the wound segmentation, which is essential for the task of evaluating the wound size and monitoring its healing. The developed wound segmentation algorithm, combining the Resent34 neural network and U-NET architecture, in combination with data augmentation, shows promising results on actual data. It can be a helpful tool in medical practice for the automatic segmentation of the wound and for evaluating its characteristics for a more accurate diagnosis and treatment management. Figures 6, 7 shows the training and validation IOU and loss, respectively.

Below is a table with the results of cross-validation conducted to assess the performance of the proposed model. Cross validation was performed using the k-fold cross-validation method on the training augmented dataset. Model performance evaluation metrics, including IOU and Dice score, are presented in Table 1.

**Table 1:** Results of cross-validation of the model based on the k-fold method.

Fold	IOU (%)	Dice score (%)
1	97.00	98.48
2	97.89	98.93
3	98.04	99.01
4	98.55	99.27
5	98.79	99.39
6	98.97	99.48
7	98.99	99.49
8	98.43	99.21
9	99.08	99.54
10	99.15	99.58
Average values	98.49	99.24

Table 1 presents the results of the model evaluation for each of the k folds, where each fold is used as a test set and the rest of the folds as a training set. For each fold, IOU and Dice scores are provided to provide information about how well the model performs segmentation on each fold.

The averages of these metrics show the overall model’s performance on the entire dataset. In this case, the average values of IOU and Dice scores are 98.49% and 99.24% respectively, which indicates a good quality of the model. Cross-validation allows you to take into account the diversity of data and check how stable and effective the model is on different data subsets.

## 5 Discussion

The segmentation of wounds is a crucial undertaking when it comes to medical imaging, which entails the identification and differentiation of wound boundaries within images. The accurate diagnosis, treatment planning, and monitoring of the healing progress are contingent upon the appropriate segmentation of wounds. One of the primary difficulties encountered in the process of wound segmentation pertains to the inherent variability observed in the visual characteristics, dimensions, and configurations of wounds. Wounds exhibit a variety of textures, colors, and depths, encompassing a spectrum that spans from minor lacerations to extensive ulcers. The presence of variability poses a challenge in the development of a universal segmentation algorithm that can effectively segment diverse types of wounds. Various methodologies have been suggested for wound segmentation, encompassing conventional machine learning methodologies, image processing techniques, and deep learning models. The conventional methods frequently depend on the manual extraction of features and the application of thresholding techniques in order to distinguish the area of the wound from the adjacent healthy tissue. Although these methods may yield satisfactory outcomes in specific scenarios, they might encounter difficulties when dealing with intricate wounds or extensive datasets. Wound segmentation has been a subject of study in the field of machine learning, with various methods such as support vector machines, random forests, and convolutional neural networks being employed for this purpose. These algorithms acquire knowledge from a dataset that has been annotated with labels, enabling them to comprehend intricate wound patterns and distinctive attributes. Among the deep learning models, convolutional neural networks (CNNs) have demonstrated considerable potential in the field of wound segmentation. Convolutional neural networks possess the ability to autonomously acquire hierarchical features from unprocessed images, thereby enabling the incorporation of both local and global information. It has been demonstrated that they exhibit a notable level of precision in the process of wound segmentation, even when confronted with the presence of noise or other artifacts. Notwithstanding the progress made in wound segmentation, there remain a number of challenges that necessitate attention and resolution. The tasks mentioned above encompass the management of diverse wound types and stages, addressing discrepancies in imaging techniques and resolutions, and enhancing the applicability of segmentation algorithms across various datasets and medical facilities. Further research and development are needed to address the challenges and enhance the accuracy and generalizability of wound segmentation algorithms. The advantages of accurate segmentation of tiny targets and its adaptive network structure are shown by the U-Net framework, which was developed in 2015 (37). The incorporation of a U-Net as a deep learning model in diverse medical applications (39–44) has served as a prominent trigger for the motivation behind this investigation. The utilization of U-Net has been widely observed in wound segmentation. Recently, numerous studies (5, 19, 30, 45–47) have endeavored to improve their methodologies by developing enhanced models that are built upon the U-Net framework. This study presents a novel framework that combines the U-Net architecture and the ResNet34 model to improve the effectiveness of image segmentation tasks. The integration of the ResNet as an encoder within the U-Net framework for image segmentation has demonstrated remarkable performance in the segmentation of brain tumors, as evidenced by the work conducted by Abousslah et al. (48). The performance mentioned above has served as a source of inspiration for us to put forth a wound segmentation framework. This framework leverages the encoding capabilities of ResNet34 as the primary encoder, while integrating the decoder architecture of UNet. The objective is to attain accurate and all-encompassing segmentation. This section will provide an analysis of the fundamental components involved in the formulation of the model, as well as an examination of its associated benefits. First and foremost, it is essential to acknowledge that the developed model demonstrated noteworthy outcomes in the task of segmenting the injured region. This observation demonstrates the efficacy of the chosen methodology and framework employed in the model. Upon analyzing the results, it was observed that the model exhibits a notable degree of accuracy and a commendable capacity to discern the affected region within the images. This tool has the potential to be a valuable resource for healthcare practitioners in the identification and management of various types of wounds. One of the primary benefits of the developed model lies in its capacity to effectively handle diverse categories of wounds and a wide range of medical images. The model effectively addresses both superficial injuries and intricate wounds. This characteristic renders it a versatile instrument for diverse medical practice scenarios.

Furthermore, the model that has been developed exhibits the capability to automate and expedite the process of segmenting areas that have been wounded. Instead of relying on manual allocation and analysis of wounds performed by medical personnel, the proposed model offers a rapid and precise means of determining the precise location of the wound. This will enable healthcare professionals to divert their attention towards other facets of treatment and enhance the overall efficacy of the procedure. This research proposes a strategy that can be applied to other wound types utilizing data and models in clinical practice. Chronic wounds like diabetic or pressure ulcers heal differently than acute wounds like surgical incisions or burns. Thus, data and models used to detect and segment wounds must account for these variances and characteristics, including wound location, shape, depth, infection, and tissue type. Adapting and generalizing the presented technique to additional wound types and animals may result in a more comprehensive and versatile wound analysis tool for clinical practice and wound care research. However, data availability and quality, wound complexity and unpredictability, and ethical and practical issues in the use of animals for wound experiments present challenges. Future research should explore and evaluate these difficulties.

### 5.1 Comparing the performance to the state-of-the-art

As part of this study, we performed wound segmentation using modern deep-learning algorithms. In our work, we set ourselves the goal of surpassing the results of previous studies and increasing the accuracy and efficiency of the segmentation process. In this section, we compare our obtained results with the results obtained by other researchers in the field of wound segmentation. To do this, we provide a table with detailed indicators of IOU and Dice scores that are used to assess the quality of segmentation. Table 2 provides a comparison of wound segmentation results between our approach and previous studies. The table allows us to analyze the advantages and limitations of our approach compared to previous work, as well as identify possible areas for improving the results. Our conclusions and recommendations can contribute to the development of wound segmentation and increase its applicability in the practice of clinical medicine.

**Table 2:** Comparison of wound segmentation results between our approach and previous studies.

Author	Techniques	Task	IOU	Dice score
Goyal et al. (15)	FCN	Segmentation	—	79.4%
Wang et al. (27)	Deep CNN	Segmentation	73.36%	—
Huang et al. (32)	Mask R-CNN	Segmentation	69.0%	
Li et al. (49)	DCNN+	Segmentation	85.88%	—
Dhane et al. (50)	Fuzzy spectral clustering	Segmentation	—	86.7%
Carrión et al. (51)	U-Net	Segmentation	93.0%	96.0%
Carrión et al. (51)	FCN-8	Segmentation	45.90%	59.22%
Carrión et al. (51)	SegNet	Segmentation	53.88%	65.69%
Our	ResNet-U-Net	Segmentation	97.25%	98.6%

### 5.2 Limitations and future scope

The limitation of the present study is the lack of explainability of the proposed hybrid model for wound segmentation. A deep model’s inexplicability severely restricts how effectively it can be used. Enhancing the model’s explainability is a viable avenue for future scope, because it will raise the model’s understanding and applicability among scientists. It is also suggested that future research concentrate on employing transformers and other deep models to increase performance. This additional avenue can potentially enhance and optimize the wound segmentation task’s performance. Furthermore, studies in this field might result in the development of hybrid model-based techniques for wound segmentation that are more precise and effective.

## 6 Conclusion

In this paper, we suggest a deep learning methodology aimed at enhancing the generalization features of wound image segmentation. Our proposed approach involves the integration of U-Net and ResNet34 architectures, resulting in a hybrid model. The empirical evidence demonstrates that the Hybrid model yields more precise segmentation outcomes, as indicated by superior scores in terms of Intersection over Union (IOU) and Dice metrics. Furthermore, the Hybrid model effectively minimizes the occurrence of incorrectly identified regions. We discovered that employing the integration of automated wound segmentation and detection improves segmentation efficiency and allows the segmentation model to generalize effectively for out-of-distribution wound images. Therefore, considering our dataset, the utilization of both U-Net and ResNet34 in the method explained offers a benefit compared to employing the algorithms individually, even when incorporating distinct post-processing procedures.

We conclude that our findings about the hybrid model can be generalized to other medical datasets characterized by diversity in cell densities. Therefore, researchers are strongly encouraged to adopt our proposed methodology for additional research in this area.

## Data availability statement

The datasets presented in this study can be found in the following online repository: https://datadryad.org/stash/dataset/. doi: 10.25338/B84W8Q.

## Ethics statement

Ethical approval was not required for the study involving animals in accordance with the local legislation and institutional requirements. Written informed consent was not required for this study in accordance with the national legislation and the institutional requirements.

## Author contributions

MA: Funding acquisition, Formal analysis, Project administration. AB: Conceptualization, Investigation, Methodology, Resources, Software, Validation, Visualization, Writing – original draft, Writing – review & editing. NS: Conceptualization, Funding acquisition, Investigation, Project administration, Resources, Supervision, Writing – original draft, Writing – review & editing. NM: Data curation, Funding acquisition, Investigation, Methodology, Project administration, Resources, Validation, Writing – original draft, Writing – review & editing. RT: Methodology, Resources, Writing – original draft, Writing – review & editing. MM: Formal analysis, Funding acquisition, Project administration, Resources, Supervision, Writing – review & editing. TN: Writing – review & editing.
